# Carbonate chemistry fitness landscapes inform diatom resilience to future perturbations

**DOI:** 10.1126/sciadv.adu8024

**Published:** 2025-09-17

**Authors:** Aaron Ferderer, Kai G. Schulz, Anusuya Willis, Kirralee G. Baker, Zanna Chase, Lennart T. Bach

**Affiliations:** ^1^ Institute for Marine and Antarctic Studies, Ecology & Biodiversity, University of Tasmania, Hobart, TAS, Australia.; ^2^ Australian National Algae Culture Collection, National Collections and Marine Infrastructure, CSIRO, Hobart, TAS, Australia.; ^3^ Faculty of Science and Engineering, Southern Cross University, Lismore, NSW, Australia.

## Abstract

Marine diatoms are an abundant and ecologically important phytoplankton group susceptible to changing environmental conditions. Currently available data assessing diatom responses focus on empirical comparisons between present-day and future conditions, rather than exploring the mechanisms driving these responses. Here, we conducted high-resolution growth experiments to map the fitness of diatoms across broad carbonate chemistry landscapes. Our results reveal species-specific carbonate chemistry niches, which can be used to predict ecological shifts between species under changing conditions driven by ocean acidification or ocean alkalinity enhancement. The results demonstrate that changes in diatom fitness are almost exclusively driven by carbon dioxide and proton concentrations, with bicarbonate exerting no discernible effect. Thus, current assumptions regarding the role of bicarbonate as a primary carbon source supporting diatom growth may be overestimated. This study presents a methodological and conceptual framework as a foundation for future studies to collate data capable of predicting species-specific responses and shifts in ecological niches driven by changes in marine carbonate chemistry.

## Teaser

Diatom growth rates are determined by concentrations of CO_2_ and H^+^ across broad carbonate chemistry landscapes.

## INTRODUCTION

Diatoms are a group of phytoplankton, comprising 40% of all known marine phytoplankton species and are responsible for ~25% of global primary production (*1*–*3*). Their cosmopolitan distribution—relatively large size, silicified cell walls, and rapid growth rates—distinguish diatoms as key contributors to the global carbon and silicon cycles (*3*–*5*). Because of their dominance in dynamic marine environments, diatoms have been touted as one of the most important taxonomic groups in regard to global ocean processes and marine food webs (*3*, *6*, *7*).

Marine carbonate chemistry is an abiotic factor known to exert considerable control over diatom and more broadly phytoplankton fitness (*8*–*10*). Carbonate chemistry conditions have changed on biologically relevant timescales and will continue to do so due to ocean acidification (OA): the influx of anthropogenic CO_2_ into the ocean that lowers sea surface pH (*11*). Furthermore, the escalating demand for atmospheric carbon dioxide removal (CDR) to keep global warming below 2°C may require the transfer of additional atmospheric CO_2_ into the ocean (*12*, *13*). Marine CDR is potentially achievable via ocean alkalinity enhancement (OAE), an anthropogenic processes aimed at increasing seawater alkalinity to store gigatonnes of CO_2_ in seawater as HCO_3_^−^ and CO_3_^2−^ [(*14*, *15*); fig. S1]. Both OA and OAE have the ability to alter seawater carbonate chemistry beyond natural variability (*11*, *15*) and are thus of ecological relevance, particularly for photosynthesising organisms such as diatoms (*16*, *17*).

Past research investigating the response of diatoms to OA suggests that they will respond positively more often than negatively (*18*). In contrast, the influence of OAE is relatively understudied (*19*–*23*). Our current understanding of the carbonate chemistry parameters (CO_2_, HCO_3_^−^, and H^+^), which may drive changes in diatom fitness, relies on the extensive investigation of the ecological and physiological effects of OA. Many of these studies used experimental designs, which compared a control with one or few treatments simulating potential OA conditions. This approach enabled the empirical determination of how phytoplankton would respond to conditions expected for ongoing OA (*24*). Although informative, these datasets constrain our ability to predict ecologically important shifts between species under carbonate chemistry conditions outside these relatively narrow niches. Moreover, this focus on unidirectional changes—namely, increases in CO_2_ and H^+^—has limited researchers’ perspectives on the complex interactions between diatoms and the marine carbonate system. To effectively project the influence of future changes in marine carbonate chemistry, frameworks that facilitate mechanistic explanations and the collation of data across broad carbonate chemistry conditions are required (*25*, *26*). Hence, this study aimed to map the fitness of ecologically important marine diatoms across a comprehensive matrix of carbonate chemistry conditions. Fitness landscapes used here encompassed past, present, and future ocean conditions including those expected under OA and OAE. Fitness landscapes were mapped over 11 levels of total alkalinity (2000 to 3350 μmol kg^−1^) and 8 levels of dissolved inorganic carbon (DIC) (1800 to 3666.67 μmol kg^−1^), for a minimum of 67 treatments per species. Using this matrix, we assessed the growth rate, dissolved silicate (DSi) uptake, biogenic silica (BSi) production, and maximum effective quantum yield in five species of locally isolated marine diatoms.

## RESULTS

Changes in carbonate chemistry across the experimental period are illustrated in the interactive figure (fig. S6), which enables individual data exploration in the online version of the article. Changes in DIC over the experimental phase remained within 12% of initial DIC values (with the exception of two outliers; 14%). Each datapoint on the interactive figure (fig. S6) shows mean values ± SD calculated from start and end measurements of total alkalinity (TA) and pH_t_. Changes in carbonate chemistry within the experimental cultures are mainly attributed to the uptake of CO_2_ and NO_3_^−^ by the diatom cultures. Cultures, which exhibited zero net growth or mortality over the experimental period, are shown in gray on the interactive figure (fig. S6) and excluded from interpretation and model fitting.

**Fig. 1.**
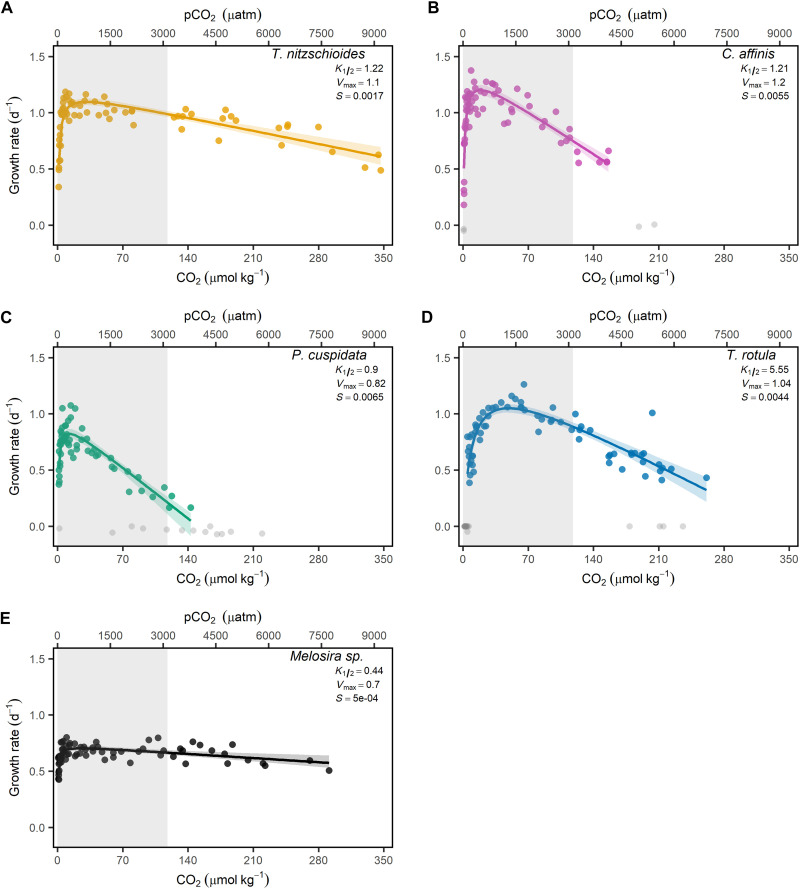
Dependence of diatom growth rates on average CO_2_/pCO_2_. Data points represent the growth of individual cultures (i.e., independent replicates) across mean CO_2_/pCO_2_ recorded within cultures at the onset and conclusion of the growth period. Gray data points indicating cultures excluded from interpretation and model calculation for (**A**) *T. nitzschioides*, (**B**) *C. affinis*, (**C**), *P. cuspidata*, (**D**) *T. rotula*, and (**E**) *Melosira* sp. The solid line indicates best fit with colored shading corresponding to the 95% confidence intervals of Eq. 1 estimated via parametric bootstrapping. The gray shaded polygon depicts CO_2_ levels used in the relevant literature (table S3).

There was no clear trend in the uptake of DSi per cell (∆DSi/cell) or cellular BSi (BSi/cell) production across species and/or carbonate chemistry conditions (interactive fig. S6). In contrast, there was a clear relationship between carbonate chemistry and *F*_v_/*F*_m_, with diatoms displaying a rapid initial increase in *F*_v_/*F*_m_ with increasing concentrations of CO_2_ and H^+^ (interactive fig. S6). Once maximum efficiencies were achieved, *F*_v_/*F*_m_ declined with continued increases in CO_2_ and H^+^ (interactive fig. S6). Variations in *F*_v_/*F*_m_ were species specific and displayed similar patterns to growth rates revealing a high correlation [*R*^2^ (coefficient of determination) ≥ 0.75] between growth and *F*_v_/*F*_m_ for all species with the exception of *Melosira* sp. (*R*^2^ = 0.53). There was a strong relationship between the growth of all species and two parameters of the marine carbonate system, namely, CO_2_ and H^+^. The model implemented here uses CO_2_ as the input variable; however, due to the strong correlation between CO_2_ and H^+^ (fig. S2) the model can be used to describe the dependence of growth on either CO_2_ or H^+^. Growth rates rapidly increased with increasing CO_2_ and H^+^, with maximum growth rates across all species observed between 13.8 (*Pseudo-nitzschia cuspidata*) (Fig. 1C) and 58.5 μmol kg^−1^ CO_2_ (*Thalassiosira rotula*) (Fig. 1D). Half saturation constants (*K*_1/2_) for *Thalassionema nitzschioides*, *Chaetoceros affinis*, and *P. cuspidata* were relatively similar and within literature values, measuring 1.22, 1.21, and 0.90, respectively (Fig. 1A and tables S1 to S3). In contrast, *T. rotula* displayed a low affinity for CO_2_ with a *K*_1/2_ of 5.55, which is notably higher than that observed in other species examined here (Fig. 1A and tables S1 and S2). Last, *Melosira* sp. displayed a high affinity for CO_2_, with a *K*_1/2_ value of 0.44, which remains within the expected range for marine diatoms (Fig. 1A and tables S1 and S2). Maximum growth rates were not sustained over a broad range of CO_2_ and H^+^, decreasing linearly with increasing concentrations after species-specific *V*_max_ values were reached. Declines in growth at elevated CO_2_ concentrations varied between species, with *Melosira* sp. displaying the lowest sensitivity (*S* = 0.0005), while *P. cuspidata* displayed the greatest (*S* = 0.0065) (Fig. 1). In contrast to the clear dependence of species’ growth rate on concentrations of CO_2_ and H^+^, no discernible trends were observed between growth rates and any other carbonate chemistry parameter (CO_3_^−2^, HCO_3_^−^, DIC, TA, or Ω_aragonite_) (interactive fig. S6).

**Fig. 2.**
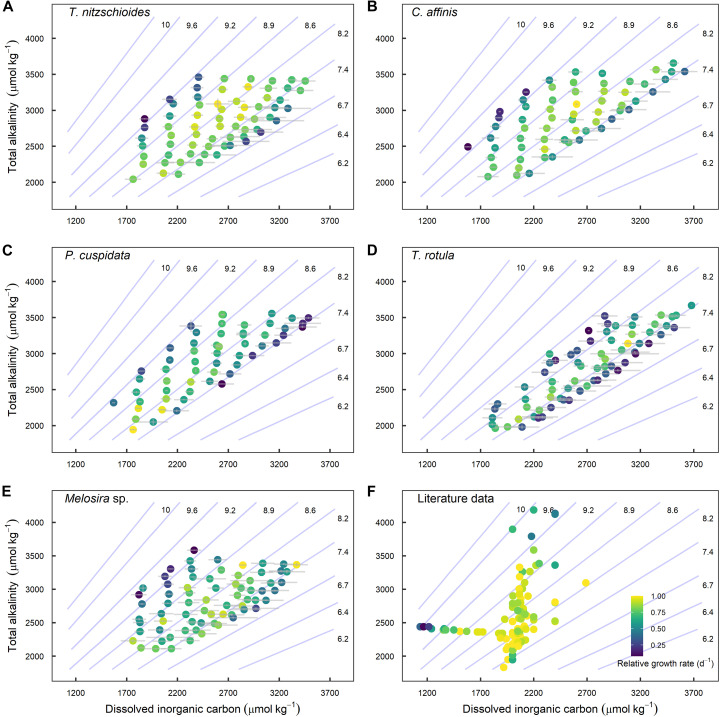
Fitness landscapes depicting changes in relative growth rate as a function of TA and DIC. Data points represent growth rates of individual cultures (i.e., independent replicates) at experimental TA and DIC for (**A**) *T. nitzschioides*, (**B**) *C. affinis*, (**C**), *P. cuspidata*, (**D**) *T. rotula*, and (**E**) *Melosira* sp., and diatom species assessed (**F**) within the literature. Isobars represent pH on the total scale according to values of DIC and TA calculated at 15°C, salinity 33, and using predefined constants (*67*). Note that one data point is excluded from plot (F) as it is far outside the axis scaling (*19*).

The application of the Eqs. 1 and 2 to the literature data revealed similar dependence of growth on CO_2_ with optimum curve responses identified over an average of three to four CO_2_ levels (fig. S3). Diatom growth increased as CO_2_ increased from 0 μmol kg^−1^ before tapering as maximum growth rates were achieved for all species (fig. S3). Although species displayed optimum curve responses, there were relatively small differences in growth across two-dimensional fitness landscapes (Fig. 2F). This is with the exception of cultures grown at the lowest CO_2_ levels illustrated by data points at low and standard DIC (2000 μmol kg^−1^) but high TA (~3500 to 4200 μmol kg^−1^) (Fig. 2F).

All species assessed here and 51% of those within the current literature conformed to an optimum curve with growth decreasing at elevated concentrations of CO_2_ and H^+^ (fig. S3). The absence of growth inhibition in some species within the literature is likely due to the relatively low maximum CO_2_ and H^+^ concentrations explored within the specific studies. This is with the exception of three species where there was no clear decrease in growth at concentrations of H^+^ exceeding 75 μmol kg^−1^ [(*27*); *R. alata* and *T. punctigera*, (*28*); *Thalassiosira pseudonana*] (fig. S3). The evaluation of the “average diatom response,” which encompasses all available growth and CO_2_ data, supports the general trend among species while also highlighting the large variation in growth rates and tolerances between diatom species at elevated CO_2_ and H^+^ concentrations (fig. S3). Similar patterns are apparent between our data and relevant literature data, with growth rates remaining relatively consistent across a range of TA from ~2000 to 3000 μmol kg^−1^ and constant DIC of ~2000 μmol kg^−1^ (Fig. 2). Note that the distinct differences in color distribution between data collected in this study and that of the relevant literature (Fig. 2). This is due to Fig. 2F displaying the growth of several diatom species with varying optimal conditions and the relatively few treatment levels within each study reducing the range of carbonate chemistry conditions at which relative growth rates are observed (fig. S4). In addition, growth rates were negatively affected above pH ~9.2 in both our data and that of the literature (Fig. 2).

## DISCUSSION

**Fig. 3.**
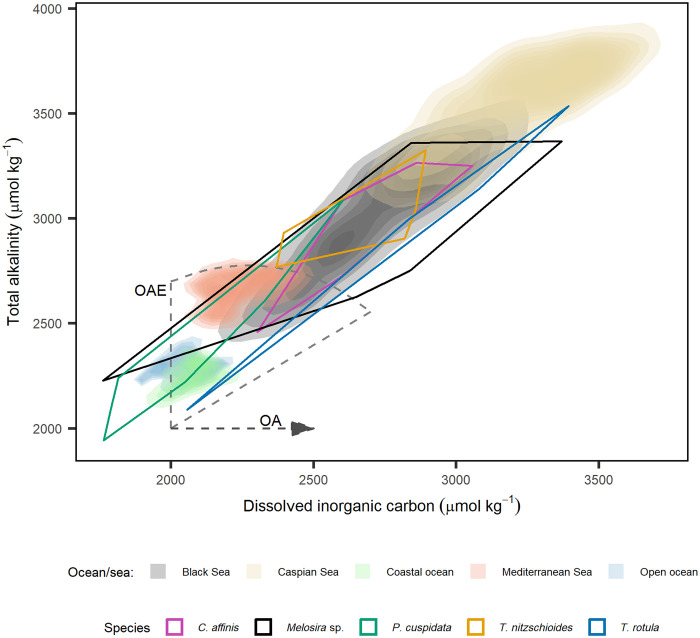
Density plot displaying current, future, and optimal carbonate chemistry conditions for marine diatoms. Density plot displaying concentrations of DIC and TA in global oceans and marginal seas with elevated alkalinity. Data for global surface oceans (<50 m) were collected from The Global Ocean Data Analysis Project (GLODAP) (*69*, *70*), and data for marginal seas were collected from The World Ocean Database (WOD) (*71*). Coastal data represents samples where the maximum depth at the sample site was <200 m and open ocean data where maximum depth was >200 m. Overlying polygons depict TA and DIC at which species-specific growth was within the 90th percentile. The horizontal dashed line indicates changes in carbonate chemistry via OA, while the fan indicates potential conditions resulting from OAE depending on the degree of air-sea CO_2_ equilibration (*21*).

Our experiments aimed to generate diatom fitness landscapes by mapping diatom growth across a broad range of carbonate chemistry conditions encompassing those that occur within the ocean, marginal seas, and under various future ocean change scenarios (Figs. 2 and 3). Fitness landscapes of the five marine diatoms assessed here enable us (i) to determine the physiological capacity of diatoms to grow under changing carbonate chemistry conditions (ii) identify species-specific carbonate chemistry niches and—most importantly—(iii) enable the exploration of the mechanistic drivers underlying the observed changes.

### Growth dependency on key carbonate chemistry parameters

Disentangling the key carbonate chemistry parameters that influence diatom fitness improves both our mechanistic understanding and ability to predict the response of diatoms to changing ocean conditions (*29*). Because of strong correlation and covariation between parameters within the marine carbonate system, it is inherently difficult to isolate those responsible for driving changes in phytoplankton growth (i.e., CO_3_^−2^, HCO_3_^−^, DIC, TA, and/or Ω_aragonite_) (*30*). However, there is evidence to suggest growth is dependent on H^+^ due to disruptions of intracellular pH homeostasis under high CO_2_/H^+^ conditions, while sub-saturating concentrations of CO_2_ are believed to drive changes in growth under low CO_2_/H^+^ conditions (*31*–*34*). Our study supports this with growth rates following an optimum curve response to changes in CO_2_/H^+^ that explain 67 to 77% of the variation in growth rates among species, except for *Melosira* sp., where they explained 45%. It is of note that, although Eq. 1 uses CO_2_ as an input value, due to the tight correlation between CO_2_ and H^+^ (fig. S2), the model results provide information on diatom growth as a result of changes in CO_2_ at sub-saturating conditions and H^+^ at inhibiting concentrations.

Diatoms are generally considered to be highly efficient in their use of CO_2_, which is supported here with species achieving maximum growth rates at relatively low concentrations of CO_2_ (4.8 to 35.1 μmol kg^−1^). Furthermore, we observed continued growth, although considerably decreased, at concentrations as low as 0.8 to 4.63 μmol kg^−1^ CO_2_. It has long been suggested that diatoms can persist in low CO_2_ environments because of their diverse and highly efficient carbon concentrating mechanisms (CCMs) which enable the use of alternative carbon sources such as HCO_3_^−^ [(*35*, *36*); fig. S5]. Current research assessing carbon uptake in marine diatoms indicates that HCO_3_^−^ and CO_2_ are equally viable sources of carbon, with diatoms regulating their uptake of HCO_3_^−^ and CO_2_ based on extracellular CO_2_ concentrations (*37*–*39*). However, we found little evidence to suggest that HCO_3_^−^ or any other carbonate chemistry parameter (TA, DIC, CO_3_^2−^, or Ω_aragonite_) had an influence on the growth rate of the diatom species assessed here. This is particularly notable given physiological research on carbon uptake has identified HCO_3_^−^ to constitute between 30 and 80% of the total carbon taken up by some species of marine diatom (*37*, *38*, *40*). This suggests that although an important source of carbon, concentrations of HCO_3_^−^ have little influence over the growth of marine diatoms, in comparison to CO_2_ and H^+^.

The seemingly limited importance of HCO_3_^−^ may be due to differences in the relative changes in carbon species concentrations. This can be illustrated by the species *C. affinis* where we observed a decrease in growth rate from 1.38 to 0.18 as CO_2_ concentrations dropped from 8.9 to 0.9 μmol kg^−1^ (Δ7.95 μmol kg^−1^). This is equivalent to an 87% decrease in growth with a 90% decrease in CO_2_ in comparison to a decrease in HCO_3_^−^ of 67% or 1280 μmol kg^−1^. Furthermore, in order for diatoms to use HCO_3_^−^, it must be converted to CO_2_, the inorganic carbon substrate of RuBisCO. This presents two issues, first, membrane bilayers are impermeable to HCO_3_^−^, and thus, active transport across membrane barriers is required (*41*). Second, CO_2_ must be actively concentrated within the cell to support photosynthesis, leading to consistently higher intracellular CO_2_ concentrations relative to the external environment. This gradient exists across a range of external CO_2_ concentrations, but under low-extracellular CO_2_ conditions, the energetic cost of maintaining it increases, with the passive flux of CO_2_ (leakage) from the cell becoming more pronounced (*35*, *42*). Although no experimental quantification of CCM energetics has been achieved, conceptual models estimate energy requirements in the operation of CCMs to be equivalent to the cost of concentrating 1 mol of CO_2_ multiplied by the ratio of CO_2_ transported for fixation to CO_2_ lost via leakage (*35*, *41*, *42*). Hence, the energetic cost of operating CCMs is primarily dependent on the ratio of transport to loss with changes in this ratio being inherently dependent on extracellular concentrations of CO_2_. Estimates provided in a recent study from carbon flux measurements in *Phaeodactylum tricornutum* suggest that the operation of CCMs can increase cellular energetic costs by 13 to 51% when compared to the minimum energy required to fix carbon via the Calvin Benson cycle (*35*). These increases in energy use for carbon acquisition would presumably impact other cellular processes such as growth. The conceptual CCM model, in combination with the results presented here, suggests that extracellular HCO_3_^−^ concentrations within the OA-OAE nexus are inconsequential for diatom growth. Instead, diatom growth appears to be dependent on extracellular concentrations of CO_2_ due to its constraint on CCM efficiency. Current assumptions regarding the importance of HCO_3_^−^ as a primary carbon source supporting diatom growth may therefore be grossly overestimated when considered under current and potential marine carbonate chemistry conditions.

### Species-specific differences

**Fig. 4.**
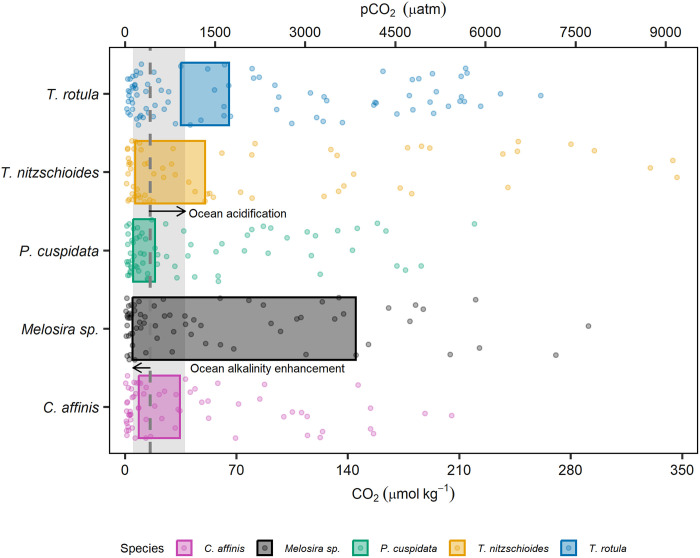
The influence of CO_2_/pCO_2_ on diatom growth rates. Fitness landscape illustrating species-specific growth rates (data points) and optima (colored rectangles), across a range of CO_2_/pCO_2_. The dashed line represents current pCO_2_ (~423 μatm) with vectors and associated gray polygon, depicting an increase in pCO_2_ up to 1000 μatm (OA) or decrease to pCO_2_ of 136 μatm (OAE, TA = Δ300 μmol kg^−1^).

We found that marine diatoms showed a universal growth response to changes in the key carbonate chemistry parameters CO_2_ and H^+^ (Fig. 1). Despite this consistent response, strong differences in sensitivities to CO_2_ and H^+^ are evident across species-specific fitness landscapes. Previous research has classified species based on their preferred uptake of CO_2_ or HCO_3_^−^ (*37*, *38*); however, the limited importance of HCO_3_^−^ for growth found here suggests that these distinctions may be less relevant. Hence, we highlight the notable differences by assessing the range of CO_2_ and H^+^ at which species are within the 90th percentile of their maximum growth rate (hereafter referred to as optimum) (Fig. 4). Most notably, *P. cuspidata* displayed a narrow optimum across changes in CO_2_ and H^+^ despite having the second highest efficiency for CO_2_ uptake (Figs. 1 and 4). This suggests that the growth of *P. cuspidata* is disproportionately controlled by changes in concentrations of H^+^, potentially due to inefficient mechanisms for maintaining pH homeostasis (Figs. 1 and 4). This observation of a narrow carbonate chemistry niche for *P. cuspidata* is supported by previous studies whereby the genus *Pseudo-nitzschia* was found to be affected by both high and low pH (*18*, *20*, *43*). In contrast, *Melosira* sp. has a much broader carbonate chemistry niche (Fig. 4). *Melosira* sp. had the lowest *K*_1/2_ values for CO_2_ and the lowest sensitivity to H^+^ (Figs. 1 and 4). We argue that this broad tolerance is likely due to its inhabitancy of both marine and freshwater environments with previous research finding freshwater and brackish diatoms to be exceptionally tolerant to changes in pH (*44*). *T. rotula* also displayed unique tolerances to changing conditions, with the lowest efficiency for CO_2_ yet relatively broad tolerance to elevated H^+^ concentrations (Figs. 1 and 4). This is supported by previous research which found elevated concentrations of pCO_2_ to result in either no or increased growth rates in *T. rotula* (*45*, *46*). Last, *C. affinis* and *T. nitzschioides* displayed similar efficiencies for CO_2_, although *T. nitzschioides* had a greater tolerance to high H^+^ concentrations suggesting the existence of efficient mechanisms for regulating intracellular H^+^ concentrations.

Diatoms are known to display species-specific optima in response to various abiotic factors with research assessing their response to temperature and salinity commonly using the prefixes eury- and steno- to define those with wide and narrow optima, respectively (*47*, *48*). These classifications present a useful format to define species, which may be at a greater or lesser risk under changing carbonate chemistry conditions. We propose that species with a narrow optimum range over changing CO_2_ and H^+^ to be classified as stenocarbs (e.g., *P. cuspidata*) while those with broad tolerances (e.g., *Melosira* sp.) classified as eurycarbs (Fig. 4). These definitions and identification of at-risk species and their thresholds in relation to CO_2_ and H^+^ are crucial, as assessments based on other carbonate chemistry parameters fail to accurately reflect the true breadth of species’ fitness optima. Mapping species optima over TA/DIC landscapes reveals this, as equivalent CO_2_/H^+^ concentrations may be achieved at multiple TA/DIC values, resulting in seemingly broader tolerances. This is illustrated by *P. cuspidata*, which exhibits a relatively narrow carbonate chemistry niche in Fig. 4 but much broader niche in Fig. 3 when compared to other species. However, decreases in CO_2_ resulting from increased TA and pH may be mitigated by concomitant increases in DIC, thus resulting in equivalent CO_2_ concentrations. The opposite is also possible, whereby a decline in DIC resulting in decreased CO_2_ can be mitigated through a concomitant decrease in TA. This highlights the importance of correctly classifying species in relation to the carbonate chemistry parameters responsible for fitness (CO_2_ and H^+^).

We found no clear trend between diatom silicification and carbonate chemistry or growth within this study. Previous research has found strong links between silicification, growth, and carbonate chemistry conditions in several species of diatom (*49*, *50*). Of particular interest are species of *Pseudo-nitzschia* with previous research finding BSi production to increase in response to OAE and decrease in response to OA (*20*, *50*). Silicification is generally linked to growth with silicification increasing at low growth due to a lengthening of the G_2_ phase where silica is incorporated into the frustule (*51*, *52*). Hence, increases in cellular BSi were expected under suboptimal carbonate chemistry conditions for growth. We suspect that the absence of such a trend may be a methodological issue within our study, with cellular debris influencing BSi measurements and resulting in suboptimal partitioning of BSi to cellular BSi concentrations. Irrespective of this, further investigation is warranted, as strong correlations between growth rates and silicification found in the literature suggest that rates of silicification may be influenced by CO_2_ and H^+^.

### Implications for anthropogenic changes in marine carbonate chemistry

Our study underlines that the growth and photosynthetic health of diatoms are most strongly influenced by concentrations of CO_2_ and H^+^, while other components of the marine carbonate system including HCO_3_^−^ appear to be of little importance. Such a limited impact of HCO_3_^−^ was unexpected and conflicts with previous work on inorganic carbon uptake, which has found it to be a major source of carbon in diatoms possessing CCMs, particularly as CO_2_ concentrations decrease (*39*, *40*). In contrast to this, diatom tolerances to changes in carbonate chemistry expected to accompany OA and OAE observed here, largely conform with those presented in the current literature (*8*, *19*, *34*). However, note that these tolerances are highly species-specific, and no single combination of carbonate chemistry conditions assessed here led to optimal growth for all five diatom species (Fig. 4).

In the context of changing oceans, our research suggests that diatoms will experience the greatest growth under CO_2_ concentrations between 5.2 and 65.3 μmol kg^−1^, equivalent to ~130 to 1756 μatm pCO_2_ (excluding min and max values of *Melosira* sp.). In the context of OA, we observe clear and species-specific thresholds at which increases in H^+^ negatively affect growth. However, the range at which growth rates remain within optimal ranges (13.2 to 52.0 nmol kg^−1^ H^+^ or 930 to 3990 μatm pCO_2_) falls within predictions for the end of this century. This is with the exception of *P. cuspidata*, which is negatively affected by increases in pCO_2_ beyond 510 μatm. This supports relevant OA research, which found diatoms to respond positively in 56% of community-based studies, while the genus *Pseudo-nitzschia* was disproportionately negatively affected by OA (*18*).

In the context of OAE, our findings suggest that marine diatoms will experience limited impacts to their growth in response to changes in CO_2_ associated with increases in TA. When alkalinity is added to seawater, there is an initial decline in the concentration of CO_2_ and H^+^, which is subsequently mitigated through the influx of atmospheric CO_2_ over months to years (*53*). Hence, OAE has the greatest potential to negatively affect diatom growth at the time of alkalinity addition and shortly after addition. Four diatom species assessed here showed declines in growth below 4.8 to 8.7 μmol kg^−1^ of CO_2_ and *T. rotula* below 35.1 μmol kg^−1^. These declines in CO_2_ occur when an alkalinity of ~76 to 632 μmol kg^−1^ is added to seawater (corresponding with pH_t_ of 7.69 to 8.49 in our experiment). These values can thus arguably be seen as species-specific thresholds beyond which OAE-induced reductions in seawater CO_2_ would cause shifts toward diatom species with greater tolerance to low CO_2_ concentrations. If we exclude *T. rotula*, which under current conditions grows outside its optimum carbonate chemistry niche (Fig. 4), we determine upper thresholds of ~260 μmol kg^−1^ for additions of alkalinity (pH_t_ = 8.26), below which minimal to no biological response is expected. While real-world OAE applications may induce larger shifts in carbonate chemistry, these changes are likely to be short-lived. In contrast, the thresholds presented here reflect more sustained conditions under which community-level shifts are unlikely to occur. Note that this threshold is calculated using mean surface ocean DIC (2030 μmol kg^−1^), TA (2300 μmol kg^−1^), and experimental temperature (15°C) and salinity (33.06). As illustrated in our study, CO_2_ and H^+^ are the primary parameters controlling growth; thus, recommended thresholds for OAE will be dependent on initial conditions that will ultimately determine the concentration of CO_2_ and H^+^ in seawater after alkalinity additions. Notably, this study quantified the carbonate chemistry niche of five coastal species, and these may be inherently more tolerant to changes in carbonate chemistry in comparison to open ocean isolates (*8*, *54*). In addition, our observations are based on a single strain of each species and therefore do not capture potential variability among various genotypes of a given species, which may, in some cases, exceed the interspecific differences observed here. Local adaptation to specific carbonate chemistry conditions has been documented in *Gephyrocapsa huxleyi*, and genotype-dependent variability has also been observed in both diatoms and coccolithophores (*55*–*57*). Thus, it is important to recognize that the interspecific differences reported here do not account for intra- or interpopulation variability, which may influence the response and ecological impacts of changing carbonate chemistry on marine diatoms. Furthermore, we used the arbitrary assumption that the 90th percentile represents the threshold at which changes to species composition would occur. However, this may vary under natural conditions and within communities, potentially being higher or lower depending on the environmental context.

Despite limitations, these findings are a step forward as they enable the quantification of diatoms based on species-specific tolerances to key carbonate chemistry parameters while also facilitating the prediction of ecologically important shifts within phytoplankton communities. The identification of these tolerances, along with the carbonate chemistry parameters responsible, was only made possible by the experimental design utilized here, which enabled the mapping of species-specific carbonate chemistry niches. Future research should, where possible, consider similar designs over scenario testing (e.g., current versus future conditions) (*8*, *25*, *26*). Adopting experimental designs as used here will enhance our mechanistic understanding of species responses to changes in marine carbonate chemistry.

## MATERIALS AND METHODS

### Isolation protocol and culture maintenance

Monospecific diatom cultures were isolated from seawater samples taken from coastal Tasmania, Australia (42.5309°S, 147.2011°E) in October 2023 and March 2024. Seawater samples were taken using a plankton net and monospecific diatom cultures isolated using micro pipetting methods (*58*). Species that were abundant in the seawater samples and/or known to be cosmopolitan in distribution were preferentially isolated due to their relevance across marine ecosystems. Please note, however, that sensitivities to carbonate chemistry can differ moderately among genotypes of a species’ population (*56*) and generally do across populations (*55*, *57*). Hence, it must be kept in mind that sensitivities to carbonate chemistry reported here for the 5 genotypes do not capture strain-specific differences that exist within a species. Isolates were identified to the species level using light and scanning electron microscopy. Monospecific cultures were maintained at 15°C, at a photosynthetic active radiation of ~96 μmol photons m^−2^ s^−1^ and a 12-hour light/12-hour dark cycle, simulating average conditions of the collection site during isolation. Cultures were grown in artificial seawater media with NaNO_3_ (48 μmol kg^−1^) and Na_2_O_2_Si, H_2_NaO_4_P (3 μmol kg^−1^), Na_2_SeO_3_ (0.01 μmol kg^−1^), sterile filtered natural seawater (2 ml kg^−1^) from the cell isolation location, and f/8 concentrations of trace metals and vitamins (*59*, *60*). Artificial seawater was prepared following established methods (*61*) with the exception of the NaHCO_3_ addition, which was supplemented with 1 M Na_2_CO_3_ and 1 M HCl (Merck) solutions to achieve a DIC of ~2100 μmol kg^−1^ and TA of ~2350 μmol kg^−1^.

### Experimental design and carbonate chemistry manipulations

Independent experiments were carried out for each diatom species: *C. affinis*, *T. nitzschioides, P. cuspidata*, *T. rotula*, and *Melosira* sp. Each experiment was conducted using dilute batch cultures (*62*) at 15°C, with light-emitting diode lights providing an average irradiance of ~96 μmol photons m^−2^ s^−1^ under a 12-hour light/12-hour dark cycle. Cultures were grown in 30-ml Nalgene polycarbonate tubes mounted on a custom plankton wheel rotating at 0.85 rpm (*63*). Light intensity was recorded as the average photon flux density measured at each position on the plankton wheel.

Each experimental run was divided into two distinct phases (acclimation and experimental phase) with slight variations to the setup of carbonate chemistry conditions between the two phases. Before the commencement of an experimental run, 10 liters of media was divided into 70, 30-ml polycarbonate tubes (culture vessels) and 70 sterile 120-ml polypropylene containers. Carbonate chemistry conditions were manipulated via additions of 1 M Na_2_CO_3_ (made fresh each day after drying Na_2_CO_3_ at 280°C for >2.5 hours) and 1 M HCl solutions to the media. All manipulations of carbonate chemistry were performed in a laminar flow hood 1 to 2 hours before inoculation with cultures. This ensured that there was sufficient time for complete homogenization of the media and acclimation to 15°C. Initially, five culture vessels were inoculated with the appropriate diatom species and placed on the plankton wheel for 1 to 2 weeks at standard carbonate chemistry conditions (DIC: 2100 μmol kg^−1^, TA: 2350 μmol kg^−1^). Once cultures reached exponential growth and detectable fluorescence, one of the five cultures was used to inoculate 70 culture tubes for the acclimation phase, with carbonate chemistry manipulated in each tube before inoculation. Once cultures had acclimated to the experimental carbonate chemistry conditions (≥four generations), they were transferred to polypropylene containers (120 ml) with media adjusted to the same carbonate chemistry conditions. Following the addition of cultures, the polypropylene containers were homogenized before being used to fill a 30-ml culture vessel, which was placed on the plankton wheel. The remaining volume was used to immediately measure pH at 15°C before filtration of the remaining media via peristaltic pump and 0.2-μm nylon syringe filter. The filtrate was divided into two containers for determination of DSi stored at −20°C (5 ml) and TA (60 ml) and stored at 4°C before analysis within 1 month of collection.

### Carbonate chemistry measurements and calculations

Quantification of the carbonate system was accomplished through the measurement of pH and TA at the beginning and end of the experimental phase. pH was measured using a Metrohm 914 pH meter and a Metrohm Aquatrode Plus and PT1000 coupled electrode. Temperature and voltage were used to calibrate pH measured at the start and end of the experimental phase to the total scale (pH_t_) using certified tris buffer as described in SOP6a (*64*). Samples for TA taken before the commencement and conclusion of each experiment were determined using a Metrohm 862 Compact Titrosampler following SOP3b (*64*). All samples were titrated using a two-step titration procedure with an initial dose of ~0.05 M HCl followed by 0.035 ml additions. TA samples taken before commencing the experimental phase were run in duplicate, while single samples for each culture vessel were evaluated at the end of the experiment due to volume restrictions. Titrations were calibrated against certified reference material (CRM batch 208) supplied by A. Dickson’s laboratory, with all resulting titration curves evaluated using the “calkulate” script within PyCO2sys (*65*). Remaining carbonate chemistry parameters were calculated from measured values of pH_t_, TA, DSi, salinity, and temperature using seacarb for R (*66*). A predefined carbonic acid dissociation constant was used (*67*) with default settings for the remaining equilibrium constants. Phosphate was not measured due to volume limitations; thus, a value of 3 μmol kg^−1^ was used. This is based on the assumption that concentrations were slightly higher at the start of experiments due to culture inoculation and lower at the end of experiments due to the uptake of phosphate. Salinity was measured in triplicate within each 10 liter batch of artificial seawater using a SonTek CastAway Conductivity–Temperature–Depth (CTD) profiler, and the average value used.

### Growth, fluorescence, and harvesting of cultures

Growth and photophysiological characteristics were monitored within culture vessels using a bench-top fast rate repetition fluorometer (FastOcean Act, Chelsea Technologies, West Molesey, UK) and the Fastpro8 system (Chelsea Technologies, West Molesey, UK). Peak excitation was recorded at 450 nm, and light intensity was applied at 1.3 × 10^22^ photons m^−2^ s^−1^. The measurement protocol was set to 16 sequences per acquisition with a 100-ms sequence interval and a 0-s acquisition pitch. In vivo chlorophyll a minimum fluorescence (used as a proxy for biomass) was measured directly inside culture vessels at the experimental temperature (15°C), ~30 min before light onset. Chlorophyll measurements were completed daily and commenced the day following culture transfer into the experimental culture vessel. Growth rates were calculated as the slope of the natural log transformed dark adapted in vivo chlorophyll a minimum fluorescence measurements. A minimum of 3 days was used for all growth rate measurements. Once a culture reached maximum allowable biomass, equivalent to a ~5 to 10% change in DIC, the culture was harvested (*62*). Before harvesting, cultures were kept on the plankton wheel for ~30 min under standard light conditions before measuring the maximum effective quantum yield (reported here as *F*_v_/*F*_m_). The recorded measurements were therefore made on light-adapted cultures, but measurements were made in the dark allowing down-regulation of rapid nonphotochemical quenching.

Harvesting cultures involved removing and fixing a 500 μl of sample with 14.3 μl of a formaldehyde/hexamine mixture and flash freezing in liquid nitrogen before storing at −80°C until analysis by means of flow cytometry. Following this, pH was measured inside the culture vessels at 15°C using the same methods described above, and the remainder of the culture gently vacuum filtered onto a 0.8-μm polycarbonate filter at ~200 mbar. The filtrate was removed and stored for analysis of DSi (−20°C) and TA (4°C) before the culture vessel, and the filtration system was thoroughly rinsed with Milli-Q, and the filter stored in a plastic petri dish at −20°C until analyzed for BSi.

### Model fitting

The response of diatom growth rates to varying carbonate chemistry conditions can be explained using a modified Michaelis Menten equation (*31*)

$$V=\frac{X*{\text{CO}}_{2}}{Y+{\text{CO}}_{2}}-S*{\text{CO}}_{2}$$ (1)

where *X* and *Y* are fit parameters that are converted into half-saturation constant (*K*_1/2_) and maximum theoretical rates at optimum carbonate chemistry conditions (*V*_max_) following mathematical procedures (*31*). *S* is a sensitivity constant that allows for a decrease in growth rate at supra saturating concentrations of CO_2_. Note that H^+^ is considered to drive the decline in growth rates (*V*) at elevated CO_2_ and H^+^, but as these parameters are closely correlated, the equation is simplified by multiplication of *S* with CO_2_ (fig. S2). Equation 1 was thus used to fit models describing the effect of CO_2_ and H^+^ on the growth rate of the five diatom species assessed here. Confidence intervals were calculated using nonparametric bootstrapping with 1000 repetitions. *X*, *Y*, and *S* values were then used to calculate values and confidence intervals of *V*_max_, *K*_1/2_, and *S*, enabling comparisons to be made between species.

### Literature data extraction and modeling

In addition to the experiments carried out here, we conducted an extraction of diatom growth rate data from the peer reviewed literature. Criteria for inclusion of growth rate data were: experiments tested elevated pH consistent with that expected for OAE; carbonate chemistry was manipulated using NaOH/HCl; the study reported cultivation temperature, and CO_2_ values, or at least two measured carbonate chemistry parameters. Only articles which included >3 different CO_2_ levels were used to model the effect of CO_2_ on growth, while only articles that reported ≥2 carbonate chemistry parameters were used to produce the literature-based fitness landscape. Comparisons between experimental data and published literature are made using relative growth rates, whereby growth under specific experimental conditions is normalized to the maximum observed rate. While this approach allows for the comparison of growth responses across different carbonate chemistry conditions, differences in experimental methodologies between studies must be carefully considered when interpreting the results.

The collated literature data assessing the response of diatom growth to changing carbonate chemistry was also fitted using Eq. 1. However, many datasets explored growth rates over a relatively narrow range (~0 to 30 μmol kg^−1^ CO_2_) and hence observed no decline in growth rates under the highest reported H^+^ concentrations. In datasets where no decline in growth was evident, a standard Michaelis Menten Eq. 2 was used

$$V=\frac{V_{\text{max}}*{\text{CO}}_{2}}{K_{1/2}+{\text{CO}}_{2}}$$ (2)

where *V* is the growth rate at a given CO_2_ concentration.

### DSi, BSi, flow cytometry, and light microscopy

Samples for the quantification of DSi were defrosted and divided into two 15-ml centrifuge tubes before being measured spectrophotometrically (*68*). Samples for BSi analysis were placed in 60-ml polypropylene vials filled with 25 ml of 0.1 M NaOH solution and digested in a water bath at 80°C for 135 min. Following this, vials were allowed to cool, before 25 ml of 0.05 M H_2_SO_4_ was added. Concentrations of BSi were then measured spectrophotometrically following the same methods used for DSi measurements.

Samples for flow cytometry were defrosted at 37°C and vortexed before ~50 μl were immediately analyzed using a CYTEK Aurora flow cytometer. Diatom cultures were distinguished from debris by encircling populations based on signal strength of the V12 (violet) fluorescence channel, forward and side scatter. Flow cytometry samples for the species *C. affinis* could not be assessed via flow cytometry, likely due to the setae of cells blocking sample aperture. Consequently, the larger species, *C. affinis*, *Melosira* sp., and *T. rotula* were counted via light microscopy. Microscopy samples were defrosted at 37°C before being vortexed and three 5- or 10-μl aliquots pipetted onto a glass microscope slide. All cells within each aliquot were then counted, and an average cell count was calculated from the three aliquots.

DSi samples taken at the start and end of the experimental phase and BSi and flow cytometry/microscopy samples taken at the end of the experimental phase were used to quantify changes in silica. The change in DSi over the experiment was normalized by cellular abundance and BSi per cell calculated as BSi divided by cell abundance at the end of the experimental phase acquired via flow cytometry or microscopy.

## Supplementary Materials

### The PDF file includes:

Figs. S1 to S5 Tables S1 to S3 Legend for fig. S6 References
