# Supplementary material for: Carbonate chemistry fitness landscapes inform diatom resilience to future perturbations
Source: Sci Adv. 2025 Sep 17;11(38):eadu8024. doi: 10.1126/sciadv.adu8024 (PMC12442879; doi:10.1126/sciadv.adu8024)
Supplement: Supplementary file 1 — Figs. S1 to S5 Tables S1 to S3 Legend for fig. S6 References [file sciadv.adu8024_sm.pdf]

Science Advances
AAAS

Supplementary Materials for

Carbonate chemistry fitness landscapes inform diatom resilience to future perturbations

Aaron Ferderer *et al.*

Corresponding author: Aaron Ferderer, aaron.ferderer@gmail.com

Sci. Adv. **11**, eadu8024 (2025)
DOI: 10.1126/sciadv.adu8024

The PDF file includes:

Figs. S1 to S5
Tables S1 to S3
Legend for fig. S6
References

Other Supplementary Material for this manuscript includes the following:

Fig. S6

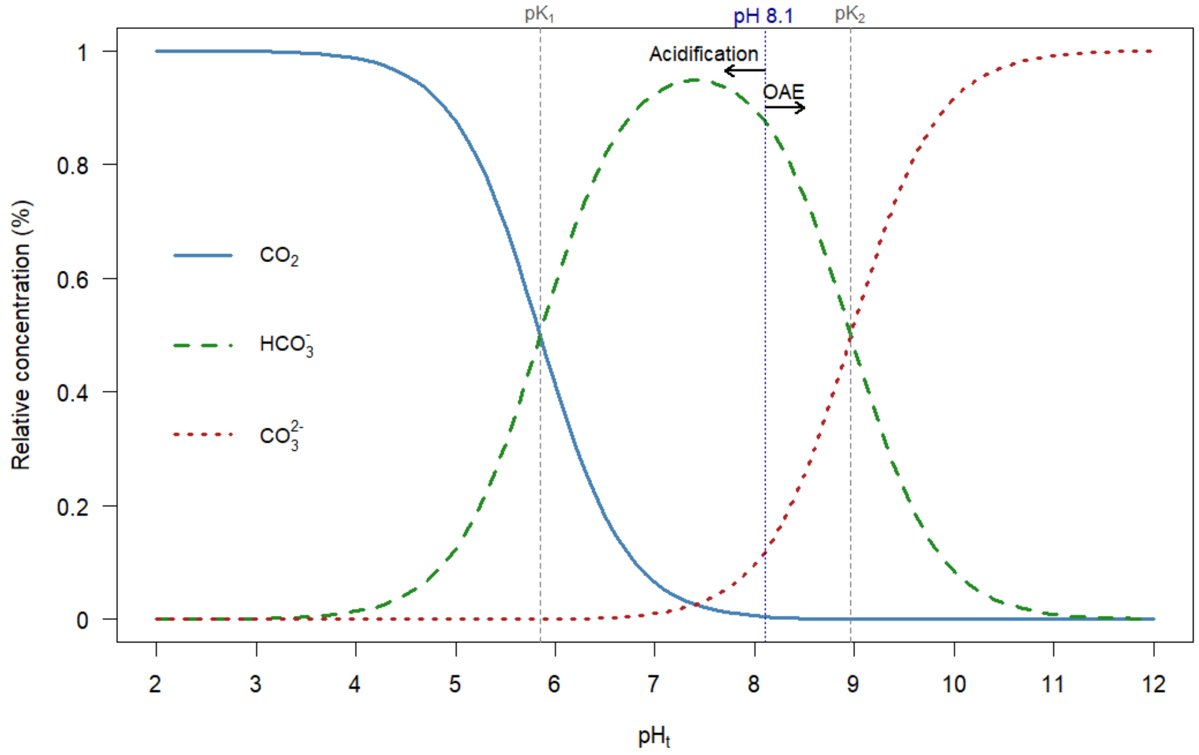

Fig. S1. Bjerrum plot illustrating relative concentrations of carbonate species (CO_2, HCO_3^-, CO_3^{2-}) as a function of pH. The figure highlights key pH values, including the pK_1 and pK_2 values for the carbonate system, and demonstrates the directional shift in carbon species under acidification and ocean alkalinity enhancement (OAE). The figure was produced using the *seacarb* package in R with default values for salinity, pressure and temperature.

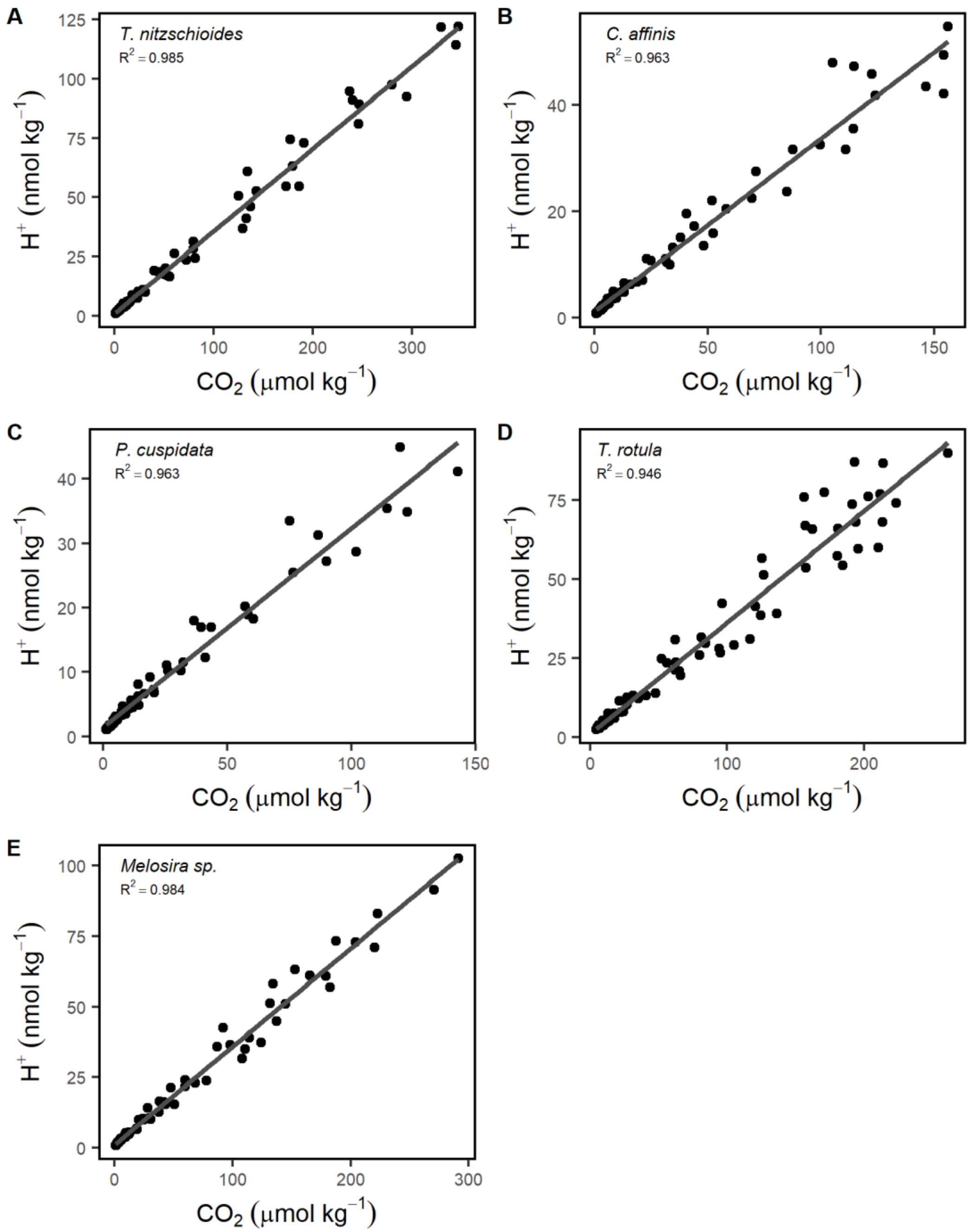

Fig. S2. Regression plots illustrating the correlation between CO_2 and H^+. Correlations between CO_2 and H^+ calculated from discrete measurements of TA and pH within cultures for; (**A**) *T. nitzschioides*, (**B**) *C. affinis*, (**C**) *P. cuspidata*, (**D**) *T. rotula*, (**E**) *Melosira sp*.

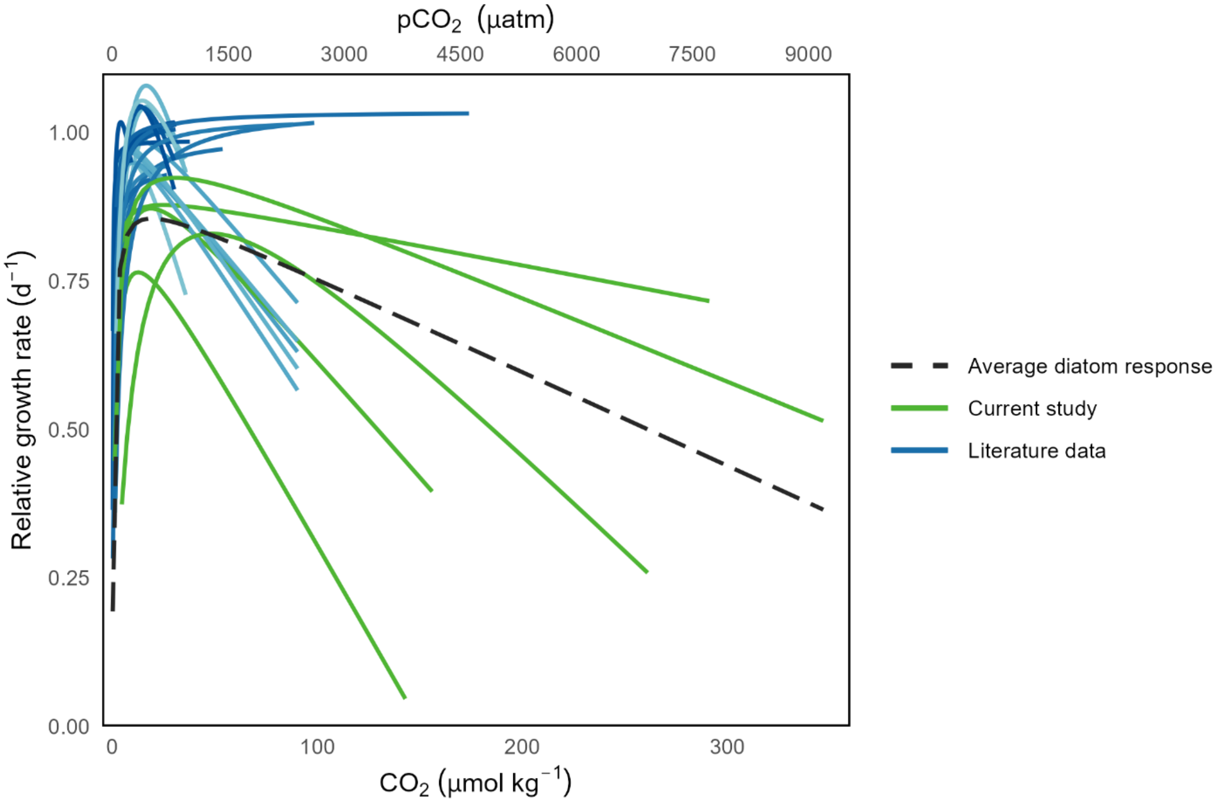

Fig. S3. Diatom growth rates as a function of CO_2. Relative growth rate of diatom species across a range of CO_2 within the current study (green) and peer-reviewed literature (Blue). Solid lines represent model fits using either equation (1) or (2) and the dashed line represents average diatom growth response.

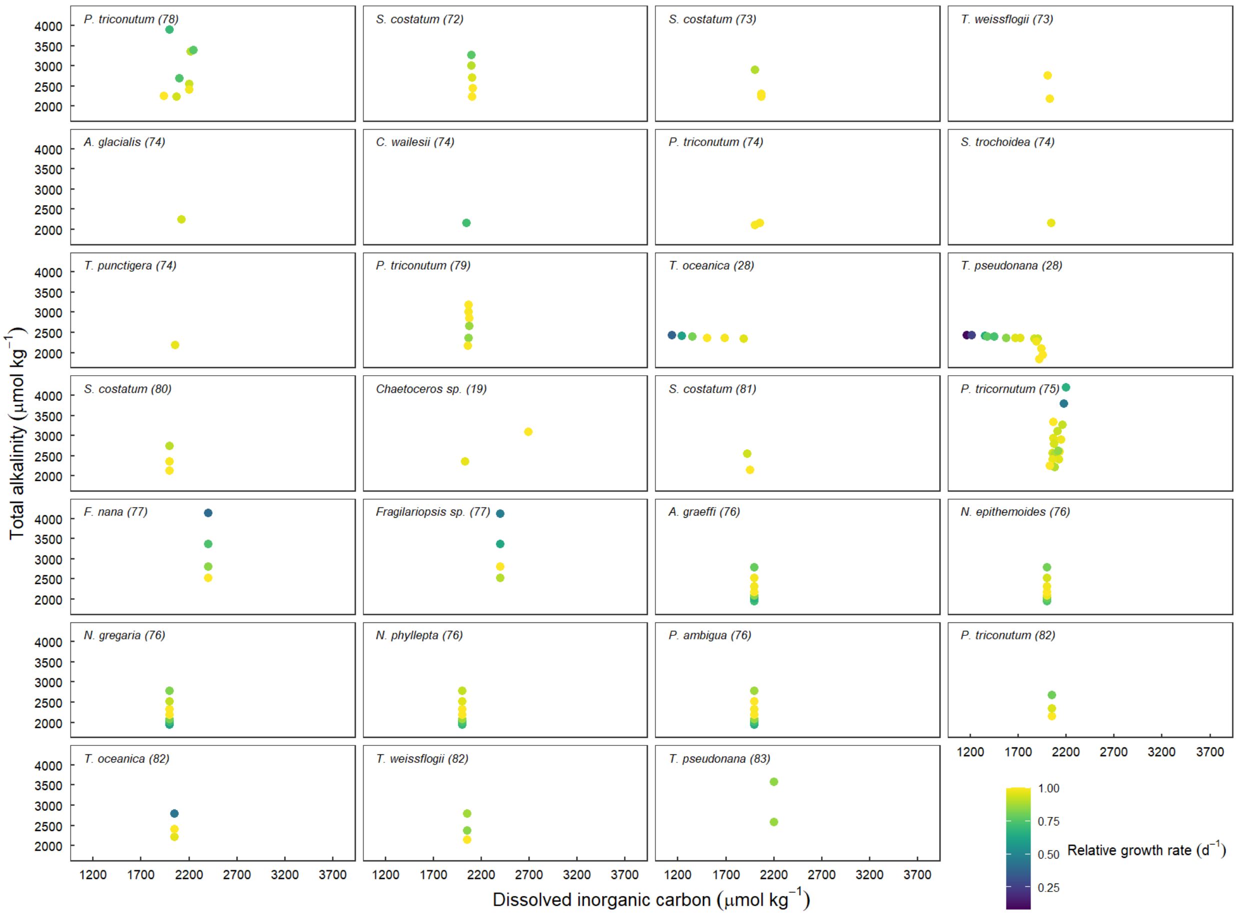

Fig S4. Relative growth rates of diatom cultures within the assessed literature. Individual panels (numbered according to the reference list) represent the growth of a given diatom culture under experimental conditions in the corresponding study. Data points represent growth rates of individual cultures (i.e., independent replicates). Note one data point is excluded from plot "*Chaetoceros* sp. (19)" as it is far outside the axis scaling (19).

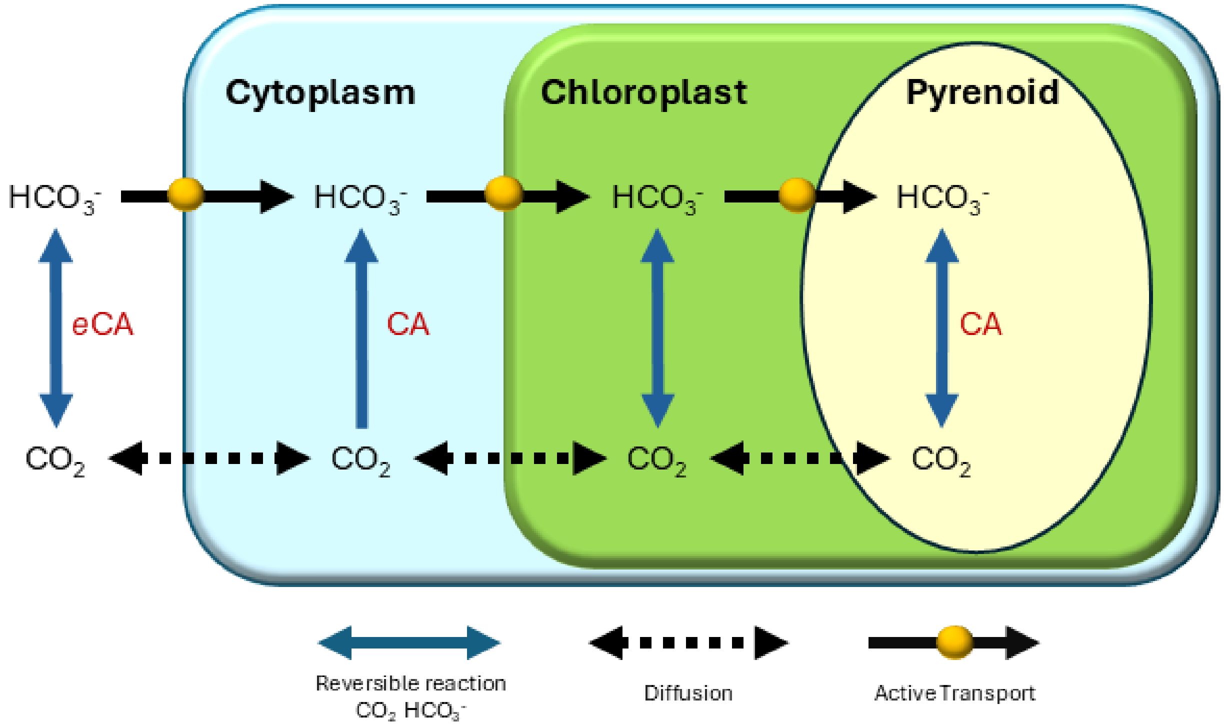

Fig. S5. Generalised diagram of a diatom carbon concentrating mechanism. Carbonic anhydrase enzymes responsible for the reversible reaction between CO_2 and HCO_3^- are represented as CA within the cell and eCA when external of the cell.

Table S1. CO_2 uptake parameters of diatom species. Half saturation ($K_{1/2}$) and maximum uptake rates (V_{max}) of CO_2 for experimental species assessed within the peer reviewed literature and current study.

Reference	Species	Treatment	V_{max} (d^{-1})	$K_{1/2}$ (µmol CO_2 kg $^{-1}$)
(72)	*S. costatum*	Control	2.17	0.48
(73)	*A. glacialis*	Light cycle 16:8	3.28	2.05
(73)	*A. glacialis*	Light cycle 24:0	1.84	1.82
(73)	*C. wailesii*	Light cycle 16:8	0.93	2.20
(73)	*C. wailesii*	Light cycle 24:0	0.38	0.83
(73)	*T. punctigera*	Light cycle 16:8	1.51	1.50
(73)	*T. punctigera*	Light cycle 24:0	0.97	1.43
(73)	*T. weissflogii*	Light cycle 24:0	1.68	0.34
(73)	*T. weissflogii*	Light cycle 8:16	1.63	1.28
(74)	*P. tricornutum*	Light cycle 16:8	1.41	0.08
(74)	*S. costatum*	Light cycle 16:8	2.18	0.65
(74)	*S. costatum*	Light cycle 24:0	1.84	0.51
(28)	*T. oceanica*	Control	0.93	0.30
(28)	*T. pseudonana*	Control	1.05	0.64
(27)	*D. brightwellii*	Control	1.44	1.18
(27)	*R. alata*	Control	0.96	2.60
(27)	*T. punctigera*	Control	1.29	1.03
(75)	*P. tricornutum*	Light level 15	0.51	0.11
(75)	*P. tricornutum*	Light level 30	1.10	0.55
(76)	*A. graeffii*	Control	0.65	0.42
(76)	*N. epithemoides*	Control	0.66	0.58
(76)	*N. gregaria*	Control	0.71	0.42
(76)	*N. phyllepta*	Control	0.73	0.19
(76)	*P. ambigua*	Control	0.68	0.30
(77)	*F. nana*	Control	0.51	0.58
(77)	*Fragilariopsis sp.*	Control	0.48	0.51
Current study	*T. nitzschioides*	Control	1.10	1.22
Current study	*C. affinis*	Control	1.20	1.21
Current study	*P. cuspidata*	Control	0.82	0.90
Current study	*T. rotula*	Control	1.04	5.55
Current study	*Melosira sp.*	Control	0.70	0.44

Table S2. CO_2 uptake parameters and associated confidence intervals. Estimated values of V_{max}, $K_{1//2}$ and S produced via nonlinear least squares models for experimental species with associated confidence intervals calculated via nonparametric bootstrapping.

Species	V_{max}			$K_{1/2}$			S		
	NLSE	Upper CI	Lower CI	NLSE	Upper CI	Lower CI	NLSE	Upper CI	Lower CI
T. nitzschioides	1.10	1.13	1.07	1.22	1.50	0.90	0.0017	0.0020	0.0013
C. affinis	1.20	1.25	1.16	1.21	1.52	0.92	0.0055	0.0063	0.0047
P. cuspidata	0.82	0.87	0.78	0.90	1.19	0.61	0.0065	0.0080	0.0057
T. rotula	1.04	1.08	1.01	5.55	6.81	4.36	0.0044	0.0052	0.0036
Melosira sp.	0.70	0.73	0.68	0.44	0.61	0.27	0.0005	0.0008	0.0003

Table S3. Literature data assessing diatom response to OAE related perturbations. Response of diatom monocultures to changes in carbonate chemistry expected under OAE within the current peer-reviewed literature. *Indicates that no salinity value was provided thus a default value of 35 was used.

Reference	Species	S	T (^{0}C)	Media	Treatment	Light (µmol m^{-2} s^{-1})	Light regime	pH range	CO_2 range
(*78*)	*P. tricornutum*	35	17.5	Enriched SW	Light level	150, 30	14:10	7.91 – 9.5	0.2 – 24.8
(*72*)	*S. costatum*	32	15	Enriched SW	C only	150	18:6	7.89 – 9.03	1.5 – 30.6
(*74*)	*A. glacialis, C. wailesii, P. tricornutum, S. costatum, T. punctigera*	31	15	Enriched SW	Light regime	150	16:8, 24:0	7.8 – 7.9*	1.45 – 37.8
(*73*)	*A. glacialis, C. wailesii, P. tricornutum, T. punctigera, T. weissflogii*	31	15	Enriched SW	Light regime	150	16:8, 24:0, 8:16	7.82 – 9.02*	1.32 – 37.7
(*79*)	*S. costatum, T. weissflogii*	31	15	Enriched SW	Light regime	150	12:12, 16:8, 24:0	7.97 – 8.87	2.6 – 25.8
(*28*)	*T. oceanica, T. pseudonana*	35	20	Enriched SW	C only	100	24:0	7.07 – 9.48	0.18 – 174
(*80*)	*S. costatum*	35*	20	Enriched SW	Light level	210,30	12:12	7.8 – 8.6	4 – 31
(*19*)	*Chaetoceros* sp.	33	15	Enriched SW	C only	170	16:8	8.24 – 8.41	10 – 11.9
(*81*)	*S. costatum*	29.4	15	Enriched SW	Low P	150	24:0	8.06 – 8.68	3.63 – 17.1
(*27*)	*D. brightwellii, R. alata, T. punctigera*	35*	17, 5	Enriched SW	C only	120	NA	NA	0.807 – 98.4
(*75*)	*P. tricornutum,*	31	15	Enriched SW	Light level	150,15,30	NA	7.9 – 9.58	0.23 – 30.2
(*77*)	*F. nana, Fragilariopsis* sp.	33	3	L1	C only	50	16:8	8 – 10	0.54 – 27.6
(*76*)	*A. graeffii, N. epithemoides, N. gregaria, N. phyllepta, P. ambigua*	30	18	Modified F/2 N:P = 17:1	C only	600	16:8	7.25 – 8.75	2.14 – 90.3
(*82*)	*P. tricornutum, T. oceanica, T. weissflogii*	35	20	Enriched SW	Fe conc.	125	24:0	7.7 – 8.6	2.95 – 30
(*83*)	*T. pseudonana*	36	20	Enriched SW	Zn conc.	500	14:10	8.2 – 9	0.9 – 10

Interactive figure S6. Interactive fitness landscape depicting changes in diatom growth as a function of each carbonate chemistry parameter.

Data points represent growth rates of individual cultures (i.e., independent replicates) across mean carbonate chemistry conditions (± standard deviation) recorded within cultures at the onset and conclusion of the growth period. Grey data points indicate cultures that were excluded from final analysis. The interactive figure (S6) can be accessed directly via the following link: https://a-ferderer.github.io/Interactive_Carbonate_Chemistry_Fitness_Landscape/

REFERENCES AND NOTES

1. C. B. Field, M. J. Behrenfeld, J. T. Randerson, P. Falkowski, Primary production of the biosphere: Integrating terrestrial and oceanic components. *Science* **281**, 237–240 (1998).

2. N. Simon, A.-L. Cras, E. Foulon, R. Lemée, Diversity and evolution of marine phytoplankton. *C. R. Biol.* **332**, 159–170 (2009).

3. P. Tréguer, C. Bowler, B. Moriceau, S. Dutkiewicz, M. Gehlen, O. Aumont, L. Bittner, R. Dugdale, Z. Finkel, D. Iudicone, O. Jahn, L. Guidi, M. Lasbleiz, K. Leblanc, M. Levy, P. Pondaven, Influence of diatom diversity on the ocean biological carbon pump. *Nat. Geosci.* **11**, 27–37 (2018).

4. E. Litchman, C. A. Klausmeier, Trait-based community ecology of phytoplankton. *Annu. Rev. Ecol. Evol. Syst.* **39**, 615–639 (2008).

5. P. J. Tréguer, C. L. De La Rocha, The World Ocean Silica Cycle. *Annu. Rev. Mar. Sci.* **5**, 477–501 (2013).

6. E. V. Armbrust, The life of diatoms in the world’s oceans. *Nature* **459**, 185–192 (2009).

7. G. Sarthou, K. R. Timmermans, S. Blain, P. Tréguer, Growth physiology and fate of diatoms in the ocean: A review. *J. Sea Res.* **53**, 25–42 (2005).

8. A. J. Paul, L. T. Bach, Universal response pattern of phytoplankton growth rates to increasing CO_2. *New Phytol.* **228**, 1710–1716 (2020).

9. B. Rost, I. Zondervan, D. Wolf-Gladrow, Sensitivity of phytoplankton to future changes in ocean carbonate chemistry: Current knowledge, contradictions and research directions. *Mar. Ecol. Prog. Ser.* **373**, 227–237 (2008).

10. K. Gao, D. A. Campbell, K. Gao, D. A. Campbell, Photophysiological responses of marine diatoms to elevated CO_2 and decreased pH: A review. *Funct. Plant Biol.* **41**, 449–459 (2014).

11. J.-P. Gattuso, L. Hansson, *Ocean Acidification* (Oxford Univ. Press, USA, 2011).

12. J. Fuhrman, H. McJeon, P. Patel, S. C. Doney, W. M. Shobe, A. F. Clarens, Food–energy–water implications of negative emissions technologies in a +1.5 °C future. *Nat. Clim. Change* **10**, 920–927 (2020).

13. A. Oschlies, L. T. Bach, R. E. M. Rickaby, T. Satterfield, R. Webb, J.-P. Gattuso, Climate targets, carbon dioxide removal, and the potential role of ocean alkalinity enhancement. *State Planet* **2-oae2023**, 1–9 (2023).

14. H. S. Kheshgi, Sequestering atmospheric carbon dioxide by increasing ocean alkalinity. *Energy* **20**, 915–922 (1995).

15. P. Renforth, G. Henderson, Assessing ocean alkalinity for carbon sequestration. *Rev. Geophys.* **55**, 636–674 (2017).

16. L. T. Bach, S. J. Gill, R. E. M. Rickaby, S. Gore, P. Renforth, CO_2 removal with enhanced weathering and ocean alkalinity enhancement: potential risks and co-benefits for marine pelagic ecosystems. *Front. Climate* **1**, 7 (2019).

17. J. M. Guinotte, V. J. Fabry, Ocean acidification and its potential effects on marine ecosystems. *Ann. N. Y. Acad. Sci.* **1134**, 320–342 (2008).

18. L. T. Bach, J. Taucher, CO_2 effects on diatoms: A synthesis of more than a decade of ocean acidification experiments with natural communities. *Ocean Sci.* **15**, 1159–1175 (2019).

19. J. A. Gately, S. M. Kim, B. Jin, M. A. Brzezinski, M. D. Iglesias-Rodriguez, Coccolithophores and diatoms resilient to ocean alkalinity enhancement: A glimpse of hope? *Sci. Adv.* **9**, eadg6066 (2023).

20. A. Ferderer, K. G. Schulz, U. Riebesell, K. G. Baker, Z. Chase, L. T. Bach, Investigating the effect of silicate- and calcium-based ocean alkalinity enhancement on diatom silicification. *Biogeosciences* **21**, 2777–2794 (2024).

21. A. Ferderer, Z. Chase, F. Kennedy, K. G. Schulz, L. T. Bach, Assessing the influence of ocean alkalinity enhancement on a coastal phytoplankton community. *Biogeosciences* **19**, 5375–5399 (2022).

22. J. A. Guo, R. F. Strzepek, K. M. Swadling, A. T. Townsend, L. T. Bach, Influence of ocean alkalinity enhancement with olivine or steel slag on a coastal plankton community in Tasmania. *EGUsphere* **21**, 2335–2354 (2023).

23. J. L. Oberlander, M. E. Burke, C. A. London, H. L. MacIntyre, Assessing the impacts of simulated ocean alkalinity enhancement on viability and growth of near-shore species of phytoplankton. *EGUsphere*, **1–21** (2024).

24. J. P. Barry, T. Tyrell, L. Hansson, G.-K. Plattner, J.-P. Gattuso, "Atmospheric CO2 targets for ocean acidification perturbation experiments," in *Guide to Best Practices for Ocean Acidification Research and Data Reporting* (Office for Official Publications of the European Communities, 2011).

25. S. Collins, H. Whittaker, M. K. Thomas, The need for unrealistic experiments in global change biology. *Curr. Opin. Microbiol.* **68**, 102151 (2022).

26. M. K. Thomas, R. Ranjan, Designing more informative multiple-driver experiments. *Ann. Rev. Mar. Sci.* **16**, 513–536 (2024).

27. U. Riebesell, D. A. Wolf-Gladrow, V. Smetacek, Carbon dioxide limitation of marine phytoplankton growth rates. *Nature* **361**, 249–251 (1993).

28. C. Y. Chen, E. G. Durbin, Effects of pH on the growth and carbon uptake of marine phytoplankton. *Mar. Ecol. Prog. Ser.* **109**, 83–94 (1994).

29. C. L. Hurd, J. Beardall, S. Comeau, C. E. Cornwall, J. N. Havenhand, P. L. Munday, L. M. Parker, J. A. Raven, C. M. McGraw, Ocean acidification as a multiple driver: How interactions between changing seawater carbonate parameters affect marine life. *Mar. Freshw. Res.* **71**, 263–274 (2019).

30. J. C. Goldman, Inorganic carbon availability and the growth of large marine diatoms. *Mar. Ecol. Progr.* **180**, 81–91 (1999).

31. L. T. Bach, U. Riebesell, K. G. Schulz, Distinguishing between the effects of ocean acidification and ocean carbonation in the coccolithophore *Emiliania huxleyi*. *Limnol. Oceanogr.* **56**, 2040–2050 (2011).

32. B. M. Hopkinson, C. Meile, C. Shen, Quantification of extracellular carbonic anhydrase activity in two marine diatoms and investigation of its role. *Plant Physiol.* **162**, 1142–1152 (2013).

33. D. M. Kottmeier, A. Chrachri, G. Langer, K. E. Helliwell, G. L. Wheeler, C. Brownlee, Reduced H^+ channel activity disrupts pH homeostasis and calcification in coccolithophores at low ocean pH. *Proc. Natl. Acad. Sci. U.S.A.* **119**, e2118009119 (2022).

34. D. Shi, H. Hong, X. Su, L. Liao, S. Chang, W. Lin, The physiological response of marine diatoms to ocean acidification: Differential roles of seawater pCO_2 and pH. *J. Phycol.* **55**, 521–533 (2019).

35. B. M. Hopkinson, C. L. Dupont, A. E. Allen, F. M. M. Morela, Efficiency of the CO_2-concentrating mechanism of diatoms. *Proc. Natl. Acad. Sci. U.S.A.* **108**, 3830–3837 (2011).

36. C. Shen, C. L. Dupont, B. M. Hopkinson, The diversity of CO_2-concentrating mechanisms in marine diatoms as inferred from their genetic content. *J. Exp. Bot.* **68**, 3937–3948 (2017).

37. S. Burkhardt, G. Amoroso, U. Riebesell, D. Sültemeyer, CO_2 and HCO_3 □ uptake in marine diatoms acclimated to different CO_2 concentrations. *Limnol. Oceanogr.* **46**, 1378–1391 (2001).

38. S. Trimborn, N. Lundholm, S. Thoms, K. U. Richter, B. Krock, P. J. Hansen, B. Rost, Inorganic carbon acquisition in potentially toxic and non-toxic diatoms: The effect of pH-induced changes in seawater carbonate chemistry. *Physiol. Plant.* **133**, 92–105 (2008).

39. S. Trimborn, D. Wolf-Gladrow, K.-U. Richter, B. Rost, The effect of pCO_2 on carbon acquisition and intracellular assimilation in four marine diatoms. *J. Exp. Mar. Biol. Ecol.* **376**, 26–36 (2009).

40. B. Rost, S. A. Kranz, K.-U. Richter, P. D. Tortell, Isotope disequilibrium and mass spectrometric studies of inorganic carbon acquisition by phytoplankton. *Limnol. Oceanogr. Meth.* **5**, 328–337 (2007).

41. J. A. Raven, J. Beardall, M. Giordano, Energy costs of carbon dioxide concentrating mechanisms in aquatic organisms. *Photosynth. Res.* **121**, 111–124 (2014).

42. A. Burlacot, G. Peltier, Energy crosstalk between photosynthesis and the algal CO_2-concentrating mechanisms. *Trends Plant Sci.* **28**, 795–807 (2023).

43. A. O. Tatters, M. Y. Roleda, A. Schnetzer, F. Fu, C. L. Hurd, P. W. Boyd, D. A. Caron, A. A. Y. Lie, L. J. Hoffmann, D. A. Hutchins, Short- and long-term conditioning of a temperate marine diatom community to acidification and warming. *Philos. Trans. R. Soc. B Biol. Sci.* **368**, 20120437 (2013).

44. B. N. Zepernick, E. R. Gann, R. M. Martin, H. L. Pound, L. E. Krausfeldt, J. D. Chaffin, S. W. Wilhelm, Elevated pH conditions associated with *Microcystis* spp. blooms decrease viability of the cultured diatom *Fragilaria crotonensis* and natural diatoms in lake erie. *Front. Microbiol.* **12**, 598736 (2021).

45. A. King, B. Jenkins, J. Wallace, Y. Liu, G. Wikfors, L. Milke, S. Meseck, Effects of CO_2 on growth rate, C:N:P, and fatty acid composition of seven marine phytoplankton species. *Mar. Ecol. Prog. Ser.* **537**, 59–69 (2015).

46. S. N. Wyatt, B. J. McNabb, D. E. Varela, Morphological and physiological responses of the cosmopolitan marine diatom *Thalassiosira rotula* to acidification. *Diatom Res.* **39**, 61–74 (2024).

47. P. W. Boyd, T. A. Rynearson, E. A. Armstrong, F. Fu, K. Hayashi, Z. Hu, D. A. Hutchins, R. M. Kudela, E. Litchman, M. R. Mulholland, U. Passow, R. F. Strzepek, K. A. Whittaker, E. Yu, M. K. Thomas, Marine phytoplankton temperature versus growth responses from polar to tropical waters – outcome of a scientific community-wide study. *PLOS ONE* **8**, e63091 (2013).

48. S. Kamakura, M. P. Ashworth, K. Yamada, D. Mikami, A. Kobayashi, M. Idei, S. Sato, Morphological plasticity in response to salinity change in the euryhaline diatom *Pleurosira laevis* (Bacillariophyta). *J. Phycol.* **58**, 631–642 (2022).

49. F. Li, J. Fan, L. Hu, J. Beardall, J. Xu, D. Fields, Physiological and biochemical responses of *Thalassiosira weissflogii* (diatom) to seawater acidification and alkalization. *ICES J. Mar. Sci.* **76**, 1850–1859 (2019).

50. K. Petrou, K. G. Baker, D. A. Nielsen, A. M. Hancock, K. G. Schulz, A. T. Davidson, Acidification diminishes diatom silica production in the Southern Ocean. *Nat. Clim. Chang.* **9**, 781–786 (2019).

51. V. Martin-Jézéquel, M. Hildebrand, M. A. Brzezinski, Silicon metabolism in diatoms: Implications for growth. *J. Phycol.* **36**, 821–840 (2000).

52. K. R. Timmermans, B. Van Der Wagt, H. J. W. De Baar, Growth rates, half-saturation constants, and silicate, nitrate, and phosphate depletion in relation to iron availability of four large, open-ocean diatoms from the Southern Ocean. *Limnol. Oceanogr.* **49**, 2141–2151 (2004).

53. J. He, M. D. Tyka, Limits and CO_2 equilibration of near-coast alkalinity enhancement. *Biogeosciences* **20**, 27–43 (2023).

54. J. J. Valenzuela, A. L. G. de Lomana, A. Lee, E. V. Armbrust, M. V. Orellana, N. S. Baliga, Ocean acidification conditions increase resilience of marine diatoms. *Nat. Commun.* **9**, 2328 (2018).

55. G. S. I. Hattich, L. Listmann, J. Raab, D. Ozod-Seradj, T. B. H. Reusch, B. Matthiessen, Inter- and intraspecific phenotypic plasticity of three phytoplankton species in response to ocean acidification. *Biol. Lett.* **13**, 20160774 (2017).

56. Y. Zhang, L. T. Bach, K. T. Lohbeck, K. G. Schulz, L. Listmann, R. Klapper, U. Riebesell, Population-specific responses in physiological rates of *Emiliania huxleyi* to a broad CO_2 range. *Biogeosciences* **15**, 3691–3701 (2018).

57. G. Langer, G. Nehrke, I. Probert, J. Ly, P. Ziveri, Strain-specific responses of *Emiliania huxleyi* to changing seawater carbonate chemistry. *Biogeosciences* **6**, 2637–2646 (2009).

58. R. A. Andersen, *Algal Culturing Techniques* (Elsevier Science, 2005).

59. R. R. L. Guillard, "Culture of Phytoplankton for Feeding Marine Invertebrates," in *Culture of Marine Invertebrate Animals: Proceedings — 1st Conference on Culture of Marine Invertebrate Animals Greenport*, W. L. Smith, M. H. Chanley, Eds. (Springer, 1975), pp. 29–60.

60. R. R. L. Guillard, J. H. Ryther, Studies of marine planktonic diatoms: I. cyclotella nana hustedt, and detonula confervacea (cleve) gran. *Can. J. Microbiol.* **8**, 229–239 (1962).

61. D. R. Kester, I. W. Duedall, D. N. Connors, R. M. Pytkowicz, Preparation of artificial seawater1. *Limnol. Oceanogr.* **12**, 176–179 (1967).

62. J. LaRoche, B. Rost, A. Engel, "Bioassays, batch culture and chemostat experimentation," in *Approaches and Tools to Manipulate the Carbonate Chemistry., Guide for Best Practices in Ocean Acidification Research and Data Reporting*, U. Riebesell, V.J. Fabry, L. Hansson, J.-P. Gattuso, Eds. (2010), pp. 81–94.

63. J. A. Guo, R. Strzepek, A. Willis, A. Ferderer, L. T. Bach, Investigating the effect of nickel concentration on phytoplankton growth to assess potential side-effects of ocean alkalinity enhancement. *Biogeosciences* **19**, 3683–3697 (2022).

64. A. G. Dickson, C. L. Sabine, J. R. Christian, *Guide to Best Practices for Ocean CO_2 Measurements.* (PICES Special Publication 3, 2007).

65. M. P. Humphreys, E. R. Lewis, J. D. Sharp, D. Pierrot, PyCO2SYS v1.8: Marine carbonate system calculations in Python. *Geosci. Model Dev.* **15**, 15–43 (2022).

66. J. Gattuso, J. Epitalon, H. Lavigne, J. Orr, B. Gentili, M. Hagens, A. Hofman, J. Mueller, A. Proye, J. Rae, K. Soetaert, Seacarb: seawater carbonate chemistry. R package version 3.3.0, (2021); https://CRAN.R-project.org/package=seacarb.

67. T. J. Lueker, A. G. Dickson, C. D. Keeling, Ocean pCO_2 calculated from dissolved inorganic carbon, alkalinity, and equations for K1 and K2: Validation based on laboratory measurements of CO_2 in gas and seawater at equilibrium. *Mar. Chem.* **70**, 105–119 (2000).

68. H. P. Hansen, F. Koroleff, "Determination of nutrients," in *Methods of Seawater Analysis* (John Wiley & Sons Ltd, 1999), pp. 159–228; https://onlinelibrary.wiley.com/doi/abs/10.1002/9783527613984.ch10.

69. R. M. Key, A. Olsen, S. van Heuven, S. K. Lauvset, A. Velo, X. Lin, C. Schirnick, A. Kozyr, T. Tanhua, M. Hoppema, S. Jutterström, R. Steinfeldt, E. Jeansson, M. Ishii, F. F. Perez, T. Suzuki, "Global Ocean Data Analysis Project, Version 2 (GLODAPv2)" (Carbon Dioxide Information Analysis Center, Oak Ridge National Laboratory, US Department of Energy, 2015); https://doi.org/10.3334/CDIAC/OTG.NDP093_GLODAPv2.

70. A. Olsen, R. M. Key, S. van Heuven, S. K. Lauvset, A. Velo, X. Lin, C. Schirnick, A. Kozyr, T. Tanhua, M. Hoppema, S. Jutterström, R. Steinfeldt, E. Jeansson, M. Ishii, F. F. Pérez, T. Suzuki, The Global Ocean Data Analysis Project version 2 (GLODAPv2) – An internally consistent data product for the world ocean. *Earth Syst. Sci. Data* **8**, 297–323 (2016).

71. T. P. Boyer, O. K. Baranova, C. Coleman, H. E. Garcia, A. Grodsky, R. A. Locarnini, A. V. Mishonov, C. R. Paver, J. R. Reagan, D. Seidov, I. V. Smolyar, K. Weathers, M. M. Zweng, A. V. Mishonov, "World Ocean Database 2018" (NOAA National Centers for Environmental Information, 2018); www.ncei.noaa.gov/sites/default/files/2020-04/wod_intro_0.pdf.

72. S. Burkhardt, U. Riebesell, CO_2 availability affects elemental composition (C:N:P) of the marine diatom Skeletonema costatum. *Mar. Ecol. Prog. Ser.* **155**, 67–76 (1997).

73. S. Burkhardt, I. Zondervan, U. Riebesell, Effect of CO_2 concentration on C:N:P ratio in marine phytoplankton: A species comparison. *Limnol. Oceanogr.* **44**, 683–690 (1999).

74. S. Burkhardt, U. Riebesell, I. Zondervan, Effects of growth rate, CO_2 concentration, and cell size on the stable carbon isotope fractionation in marine phytoplankton. *Geochim. Cosmochim. Acta* **63**, 3729–3741 (1999).

75. U. Riebesell, S. Burkhardt, A. Dauelsberg, B. Kroon, Carbon isotope fractionation by a marine diatom: Dependence on the growth-rate-limiting resource. *Mar. Ecol. Prog. Ser.* **193**, 295–303 (2000).

76. B. Scholz, Effects of varying pH on the growth and physiology of five marine microphytobenthic diatoms isolated from the Solthörn tidal flat (southern North Sea, Germany). *Phycologia* **53**, 252–264 (2014).

77. D. H. Søgaard, P. J. Hansen, S. Rysgaard, R. N. Glud, Growth limitation of three Arctic sea ice algal species: Effects of salinity, pH, and inorganic carbon availability. *Polar Biol* **34**, 1157–1165 (2011).

78. A. Bartual, J. A. Gálvez, Growth and biochemical composition of the diatom phaeodactylum tricornutum at different pH and inorganic carbon levels under saturating and subsaturating light regimes. *Botanica Marina* **45**, 491–501 (2002).

79. S. Burkhardt, U. Riebesell, I. Zondervan, Stable carbon isotope fractionation by marine phytoplankton in response to daylength, growth rate, and CO_2 availability. *Mar. Ecol. Prog. Ser.* **184**, 31–41 (1999).

80. X. Chen, K. Gao, Effect of CO_2 concentrations on the activity of photosynthetic CO_2 fixation and extracelluar carbonic anhydrase in the marine diatom Skeletonema costatum. *Chin. Sci. Bull.* **48**, 2616–2620 (2003).

81. F. Gervais, U. Riebesell, Effect of phosphorus limitation on elemental composition and stable carbon isotope fractionation in a marine diatom growing under different CO_2 concentrations. *Limnol. Oceanogr.* **46**, 497–504 (2001).

82. D. Shi, Y. Xu, B. M. Hopkinson, F. M. M. Morel, Effect of ocean acidification on iron availability to marine phytoplankton. *Science* **327**, 676–679 (2010).

83. W. G. Sunda, S. A. Huntsman, Effect of CO_2 supply and demand on zinc uptake and growth limitation in a coastal diatom. *Limnol. Oceanogr.* **50**, 1181–1192 (2005).
