# Supplementary material for: Carbonate chemistry fitness landscapes inform diatom resilience to future perturbations
Source: Sci Adv. 2025 Sep 17;11(38):eadu8024. doi: 10.1126/sciadv.adu8024 (PMC12442879; doi:10.1126/sciadv.adu8024)
Supplement: Supplementary file 2 — Fig. S6 [file sciadv.adu8024_fig_s6.zip › sciadv.adu8024_fig_s6.html]

Dynamic Scatter Plot with Error Bars


Species:

X-Axis:

Y-Axis:

Z-Axis:

None
Color:

**Interactive figure.** To explore the figure, select the desired species or "All species" from the Species dropdown menu, followed by the parameters you wish to explore. Note that data can be viewed in 3D by selecting a parameter for the Z-Axis or in 2D by selecting "None" in the Z-Axis. All carbonate chemistry values are reported as the mean of the initial and end measurements ± the standard deviation. Error bars for DSi represent standard error of duplicate measurements. Cultures in grey are those which did not display net positive growth and/or did not reach target biomass during the experimental period.
